# Apolipoprotein A-I mimetic peptides (ApoAI MP) improve oxidative stress and inflammatory responses in Parkinson’s disease mice

**DOI:** 10.3389/fphar.2022.966232

**Published:** 2022-08-19

**Authors:** Hongfang Jiang, Xue Bai

**Affiliations:** Department of Gerontology and Geriatrics, Shengjing Hospital of China Medical University, Shenyang, China

**Keywords:** anti-inflammatory, antioxidant, apolipoprotein A-I mimetic peptides, Parkinson’s disease, neurotransmitters

## Abstract

**Purpose:** Parkinson’s disease (PD) is closely associated with oxidative stress and inflammatory situation. Apolipoprotein A-I mimetic peptides (ApoAI MP) have antioxidant and anti-inflammatory properties. We aimed to study the therapeutic effect of ApoAI MP on PD mice, and to explore the related mechanisms.

**Methods:** PD mice were induced by using 1-methyl-4-phenyl-1,2,3,6-tetrathydropyridine (MPTP). The model mice were treated with different concentrations of ApoAI MP. The open-field behavioral test assesses the total distance moved, the rest time, and the number of crossings and Rota-rod was used to evaluate motor coordination. Oxidative stress was identified by measuring the levels of superoxide dismutase (SOD), catalase (CAT), glutathionperoxidase (GSH-Px), malondialdehyde, ROS and H_2_O_2_. Inflammatory situation was analyzed by measuring the levels of tumor necrosis factor-α (TNF-α), interleukin-1β (IL-1β) and interleukin-6 (IL-6). Meanwhile, the scavenging activities of ApoAI MP for ABTS, DPPH, hydroxyl radical and superoxide anion, and the effects of the peptide on neurotransmitters were evaluated.

**Results:** PD model establishment increased oxidative stress and inflammatory status by increasing the concentrations of ROS and H_2_O_2_ production, and the levels of TNF-α, IL-1β and IL-6 (*p* < 0.05). ApoAI MP intervention improved PD symptoms by reducing the total moved distance and the number of passes (*p* < 0.01), and the falling times from Rota-rod, and increasing rest time (*p* < 0.05). ApoAI MP increased antioxidant properties by increasing the activities of SOD, CAT and GSH-Px, and reducing MDA concentration (*p* < 0.05). ApoAI MP addition reduced oxidative stress by scavenging ABTS, DPPH, hydroxyl radicals and superoxide anion and reducing the concentrations of ROS and H_2_O_2_ production (*p* < 0.05). ApoAI MP treatment increased anti-inflammatory capacities by reducing the concentrations of TNF-α, IL-1β and IL-6 (*p* < 0.05). HPLC analysis showed that the peptide treatment improved neurotransmitters.

**Conclusion:** ApoAI MP can improve the behavioral performance of PD mice by improving antioxidant and anti-inflammatory capacities.

## Introduction

Parkinson’s disease (PD), a neurodegenerative disease of the elderly population with motor symptoms, is the second most common chronic neurodegenerative disease after Alzheimer’s disease (AD) (Del Din et al., 2021). PD pathogenesis is closely related to many factors, such as environmental factors, genetic factors, aging and their interplay (Pang et al., 2019). The people aged ≥65 years have the high incidence of PD risk (1,500 out of 100,000 people) (Liu et al., 2021a), and the incidence of males is higher than that of females (Rocca, 2018). The clinical symptoms of PD include motor symptoms (resting tremor, rigidity, bradykinesia, etc.) and non-motor symptoms (sensory disturbance, sleep disturbance, mental disturbance, etc.) (Green et al., 2019). In recent years, the incidence has an increasing trend and a younger population (Rajan et al., 2020).

The main pathological feature of PD is the loss of dopamine (DA) neurons in the substantia nigra pars compacta (Masato et al., 2019), and the main clinical manifestations are progressive motor and behavioral disturbances (Papagno and Trojano, 2018). DA is an important neurotransmitter in the brain (Beitollahi et al., 2019). After the deformation of DA neurons, the content of DA in the striatum changes accordingly. The levels of glutathione peroxidase (GSH-Px) and GSH reduce during DA metabolism, and the oxidative stress of DA metabolism will increase (Zhang et al., 2018). Under the physiological conditions of body metabolism, a certain amount of ROS can be synthesized and released, and cause oxidative damage. Metal ions (Tosato and Di Marco, 2019), uric acid (Huang et al., 2018), ROS (Liu et al., 2021b), oxidative stress (Trist et al., 2019), and mitochondrial dysfunction (Park et al., 2018), etc., are related to PD. ROS and malondialdehyde (MDA) (Liu et al., 2021c) can cause the oxidative stress damage for PD development (Weng et al., 2018).

The mitochondrial respiratory chain has a greater impact on biological oxidation. If the structure of the mitochondrial respiratory chain is damaged, the free radicals produced by the patients will increase, which will inhibit the formation of mitochondrial complex I. In that case, ROS can increase the emergence of hydrogen peroxide (Piao et al., 2020). The activity of complex I was decreased in PD substantia nigra, which was closely related to aging-related protein DJ-1 and mitochondrial dysfunction (Franco-Iborra et al., 2018). *α*-Synuclein is the main component of Lewy bodies, which can increase the level of ROS in DA neurons (Bridi and Hirth, 2018). Inflammatory response and oxidative stress have a great influence on substantia nigra DA neurons, PD substantia nigra DA neurons have inflammatory responses (Cai et al., 2019). Microglia are toxic to DA neurons, and the reason is related to oxidative stress, and more free radicals will appear after activation (Sun et al., 2018). TNF-α and IL-1 *ß* are potential targets of neuroinflammation in PD patients (Schaaf, 2021). Evaluated levels of plasma IL-6 are closely associated with the severity of motor and non-motor symptoms in PD (Green et al., 2019).

Therefore, it is very important to prevent the PD risk with effective antioxidant and anti-inflammatory drugs. Apolipoprotein AI (ApoAI), as the main structural protein of high-density lipoprotein (HDL), is a remarkable biological indicator. It plays an increasingly important role in the diagnosis, disease assessment and prognosis of various neurological diseases. ApoAI exerts anti-atherosclerotic, anti-inflammatory and antioxidant properties (Zuin et al., 2021). ApoAI can inhibit the activation of monocytes mediated by T cells (Shavva et al., 2018), and can bind bacterial lipopolysaccharide to inhibit the production of IL-6 and TNF-α (Stamatikos et al., 2019). Apolipoprotein AI-mimetic peptides (ApoAI MP) (Ac-DWFKAFYDKVAEKFKEAF-NH2) is an 18-amino acid mimetic peptide of ApoAI and contains an amphipathic helix with a polar and a non-polar face that binds lipids. ApoAI MP are regarded to have antioxidant and anti-inflammatory properties and prevent oxidative-induced damage (White et al., 2019) and inflammatory injury (McGrath et al., 2020).

1-methyl-4-phenyl-1,2,3,6-tetrahydropyridine (MPTP) can induce acute tremor, bradykinesia, and gait disturbance. C57BL/6 is sensitive to MPTP and can mimic the pathological features of PD, so MPTP-treated C57BL/6 mice are the most widely used animal models in PD research (Han et al., 2018). This study explored the effect of novel ApoAI MP on PD and its protective mechanisms.

## Materials and methods

### Experimental animals

Forty C57BL/6 mice, 8–10 weeks, 18–22 g were purchased from Changzhou Cavens Laboratory Animal Co., Ltd. (Changzhou, China). All animals were housed in a room with constant temperature (22 ± 1°C), humidity (55%), and a light/dark cycle (12/12 h). The mice had free access to standard food and water *ad libitum*. All experiments were carried out based on the European Guideline for animal experiments 2010/63/EU (Gyger et al., 2019). All animals were adaptively raised in the animal room for one week before the experiment. All experimental processes were approved by the animal research committee of our unit.

### Reagents

ApoAI MP (DWFKAFYDKVAEKFKEAF) was synthesized by Chang Zhou KL Biotech Ltd. (Changzhou, China). 1-Methyl-4-phenyl-1,2,3,6-tetrahydropyridine hydrochloride (MPTP, CAS Number: 28,289–54–5) was purchased from Sigma-Aldrich (Shanghai, China). Superoxide dismutase (SOD, Cat. No. D799593), catalase (CAT, Cat. No. D799598), glutathione peroxidase (GSH-Px Cat. No. D799613), malondialdehyde (MDA, Cat. No. D799761), H_2_O_2_ kits (Cat. No. D799773), tumor necrosis factor-α (TNF-α, Cat. No. D721150), interleukin-1β (IL-1β, Cat. No. D721017) and interleukin-6 (IL-6, Cat. No. D721002) ELISA kits were purchased from Shanghai Shenggong Biotechnology Co., Ltd. (Shanghai, China). Total cholesterol (TC, Cat. No. D799800), and triglyceride (TG, Cat. No. D799795) kits were from Shanghai Shenggong Biotechnology Co., Ltd. (Shanghai, China). LDL cholesterol kit (LDL-c, Cat. No. XG-S64658) was from Shanghai Xige Biotechnology Company (Shanghai, China) and HDL-cholesterol kit (HDL-c, Cat. No. BK-E63491) was from Shanghai Mianke Biotechnology Company (Shanghai, China). HDL and LDL quantitation kits were purchased from Sigma (Sigma Technology, Shanghai, China, Cat. No. MAK045).

### Preparation and grouping of Parkinson’s disease models

**FIGURE 1:**
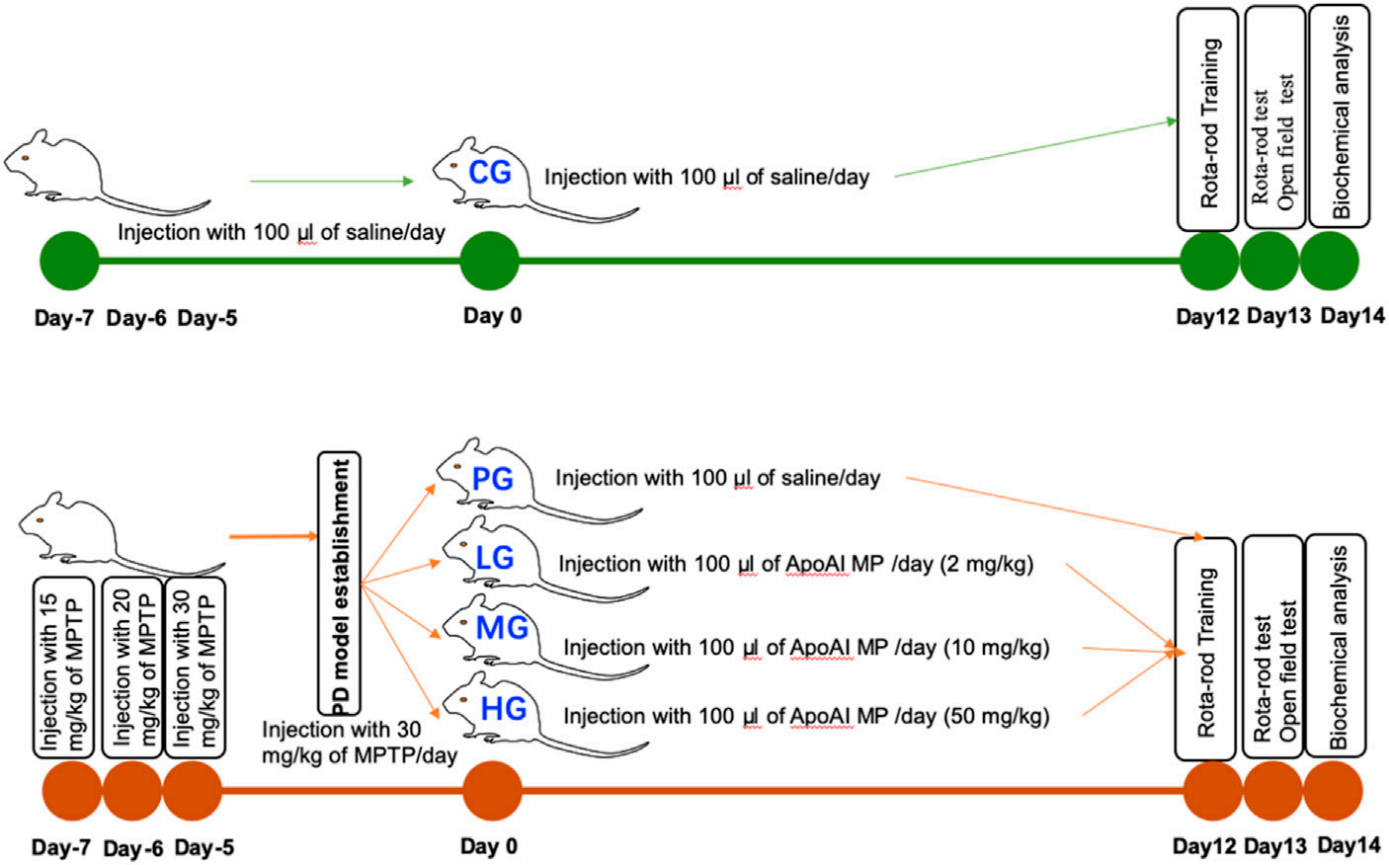
The study design of the present manuscript. Forty male mice were randomly divided into the control group (CG), the PD-model group (PG), low-dose ApoAI MP-treated group (20 mg/kg, LG), medium-dose ApoAI MP-treated group (100 mg/kg, MG), high-dose ApoAI MP-treated group (500 mg/kg, HG), and 8 mice in each group.

Forty male mice were randomly divided into the control group (CG), the PD-model group (PG), low-dose ApoAI MP-treated group (20 mg/kg, LG), medium-dose ApoAI MP-treated group (100 mg/kg, MG), high-dose ApoAI MP-treated group (500 mg/kg, HG), and 8 mice in each group. Both the PG group and the ApoAI MP group were intraperitoneally injected with MPTP to establish a PD model in mice. The dose on the first day was 15 mg/kg, the second day was 20 mg/kg; and the dose on the 3rd to 7th days was 30 mg/kg. The CG group was given an equal volume of normal saline for 7 days. The ApoAI MP group was intraperitoneally injected with 100 μl ApoAI MP with different concentrations after MPTP treatment, and the continuous administration time was 14 days (Figure 1). The CG group and the PG group were given the same volume of normal saline for 14 days (Figure 1).

### Open field behavioral test

OFBT was used to evaluate the autonomous mobility of mice. The mice were put into observation boxes (length 50 cm, width 50 cm, and height 50 cm) to adapt for half an hour; Meanwhile, the bottom of the open field was divided into 9 squares grids and OFBT was analyzed by using Smart3.0 software. After the adaptation on day 12 after PD model establishment, the environment was kept quiet, and the autonomous activities of the mice were recorded by the camera. The recording time was 10 min each time, and the video was recorded 3 times. The total path length, the resting time, and the number of crossings of the boxes, were collected and analyzed on day 13 after PD model establishment (Figure 1).

### Rota-rod test

The mice were trained for one hour before the Rota-rod test on a rotating rod (6 cm in diameter, 6 rpm) on Day 12 after PD model establishment. All mice were placed on the rotating rod and observed for two min. Rota-rod test was carried out on Day 13 after PD model establishment. The number of the mice that fell from the Rota-rod within 2 min was recorded (Figure 1).

### Grip strength test

Forelimb grip strength was measured using a grip strength meter (YLS-13A, Youcheng Jiaye Biotechnology Ltd. CO, Beijing, China). The mice to grab a horizontal bar connected with the gauge via their front paws, and their tails were gradually drawn back. Grip strength was recorded when the mouse released the bar. The test was replicated in triplicate, and the grip strength was calculated. Lower values implied that the mic had weaker muscles.

### Plasma lipids profiles assay

All the mice were fasted for 24 h before measurement on day 14 after PD model establishment. Blood samples were drawn from the tail on vena caudalis. TC, TG, LDL-c and HDL-c, LDL and HDL were measured by the above corresponding assay kits according to manufacture’s instructions. Meanwhile, the weight of all mice was measured each day.

### Measurement of DPPH (2,2-diphenyl-1-picrylhydrazyl) free radical scavenging rate

After general anesthesia on day 14 after PD model establishment, 200 mg of brain tissue specimens were collected aseptically, and liquid nitrogen was added. The tissues were ground into powder at low temperature. The powder was quickly transferred to a centrifuge tube (pre-cooled by liquid nitrogen), and 2 ml of buffer (50 mM Tris-HCl, 50 mM NaCl, pH8.0) was added. The mixture was centrifuged at 4°C, 12,000 *g* for 10 min, and the supernatant was taken for the subsequent experiments. 0.1 mM DPPH-methanol solution (reaction start tube) and pure methanol solution (un-initiative tube) were mixed with equal volume of 200 μl solution respectively. The mixture was shaken well and stood in the dark for 30 min. The absorbing values of reaction solution were measured at 520 nm. The scavenging rate of DPPH free radicals was calculated according to the Equation 1: S% = A0-(Ai-Ai0) ×A0×100% 1) where A0, Ai0, and Ai are the absorbing values of the distilled water blank group, the reaction-un-initiative tube and the reaction-started tube, respectively.

### Measurement of ABTS (2, 2′‐azinobis (3‐ethylbenzothiazoline‐6‐sulfonic acid)) free radical scavenging rate

Seven mM ABTS solution and the 2.5 mM potassium persulfate solution were mixed in equal volumes, placed at room temperature in the dark for 12 h, and then diluted to 0.7 mM with a pH 7.5 PBS solution. 800 µl ABTS was mixed with 200 µl sample in PBS solution. The absorbing values were measured at 730 nm, according to the above Equation 1 and the scavenging rate of ABTS free radicals was calculated.

### Measurement of superoxide anion scavenging rate

One hundred μL of 0.8 mM NADH and 700 μl of 10 mM Tris-HCl buffer (pH 8.0) was mixed as a reaction uninitiated tube. One hundred μL of 0.8 mM NADH, and 100 μl of 0.5 mM nitrotetrazolium chloride (NBT) and 600 μl of 10 mM Tris-HCl buffer (pH 8.0) were mixed as the reaction initiative tube. One hundred μl of the sample solution was added to all the tubes. One mM phenazine methyl sulfate (PMS) was used. After the reaction was completed, its absorbance was measured at a wavelength of 560 nm, and the scavenging rate of superoxide anion radicals was calculated according to the above Equation 1.

### Measurement of hydroxyl radical scavenging rate

Three hundred μL of 9 mM FeSO_4_ solution and 300 μL of 8 mM H_2_O_2_ were sequentially added to the 1 ml sample to initiate the reaction as a reaction initiation tube, only 300 μL of 9 mM FeSO_4_ solution was added, and no H_2_O_2_ was added as a reaction non-initiated tube. All test tubes were shaken and allowed to stand. Then 300 μl of 9 mM salicylic acid ethanol solution was prepared by adding 50% ethanol to all test tubes, reacted in a water bath at 37°C for 30 min, cool to room temperature, and measured at 510 nm. Scavenging rate of hydroxyl radicals was calculated according to the above Equation 1.

### Measurement of antioxidants

The activities of SOD, CAT and GSH-Px, and MDA concentration were measured by using SOD, CAT, GSH-Px, and MDA kits; SOD activity was measured by xanthine oxidase method (hydroxylamine method), CAT and GSH-Px activities were measured by visible spectrophotometry, and MDA content was measured by thiobarbituric acid method.

### Measurement of inflammatory factors

The levels of TNF-α, IL-1β and IL-6 were measured by using the corresponding ELISA kits according to manufacturer’s instruction. The concentration of each sample was calculated according to the standard curve of each inflammatory factor.

### Measurement of neurotransmitters

Neurotransmitters were measured according to a previous report (Zhou et al., 2019). High-performance liquid chromatography (HPLC, with fluorescence detection) was used to measure the levels of striatal dopamine (DA), 5-HT, and their metabolites, including 3, 4-dihydroxyphenylacetic (DOPAC), homovanillic acid (HVA), and 5-hydroxyindoleacetic acid (5-HIAA). The separation system was used with a T3 column (150 mm × 4.6 mm id, 5 μm). The mobile phases were water, acetonitrile, and 100 mM PBS (pH 4.0). Striatum was homogenized in 100 mM perchloric acid (10 μl/mg of striatum tissue) *via* ultrasonication centrifuged at 12,000*g*, 4°C for 10 min. Supernatant was filtered through a 0.22-μm filter and 10 μl of liquid was injected into the column. Parallelly, DA, 5-HT and their metabolites standard (Sigma, St. Louis, MO, United States) solution were prepared by using the mobile phase. The concentration of neurotransmitters was confirmed according to standard solution.

### Statistical analysis

Statistical analysis of data was performed using GraphPad Prism 8.0 software. Measured data were represented as the mean ± standard error of the mean (SEM), and one-way ANOVA was used for comparing the variable values between the two groups. Outliers were identified by using a ROUT method via GraphPad Prism Statistics Guide. *p* < 0.05 indicated that the difference was statistically significant.

## Results

### Apolipoprotein A-I mimetic peptides improved Parkinson’s disease mice open field behavior

**FIGURE 2:**
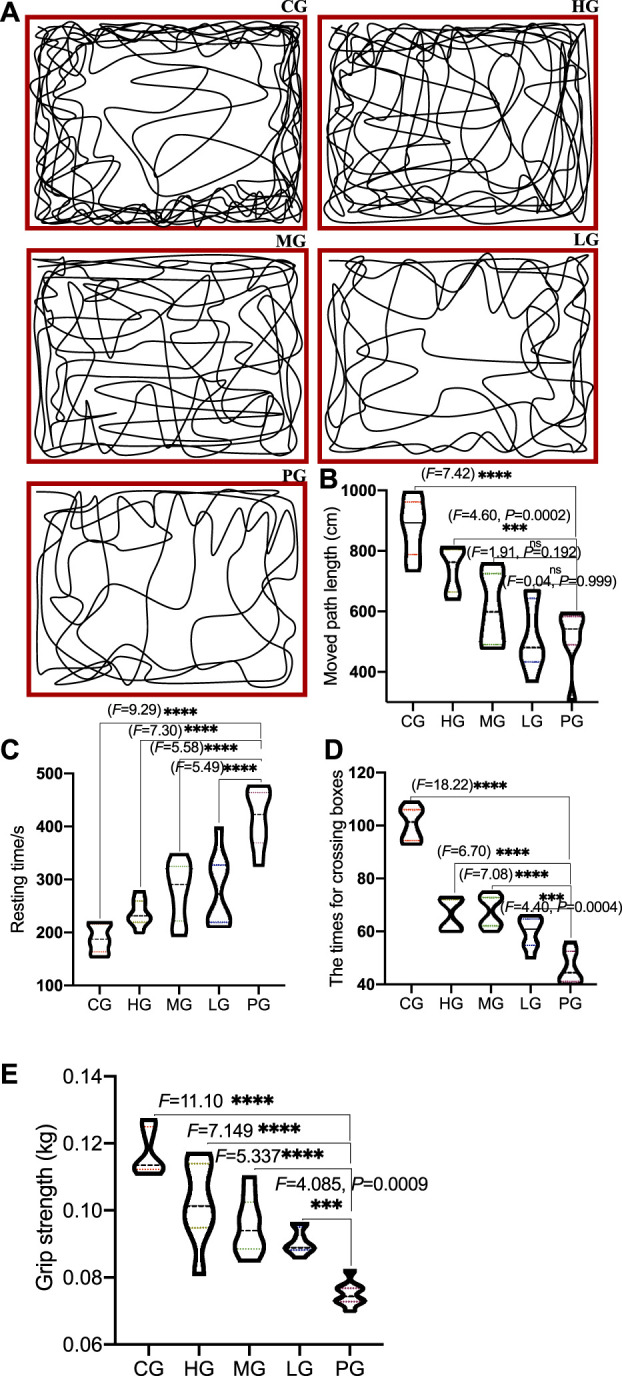
The effects of ApoAI MP on open field behavior of PD mice. **(A)** representative traces of mouse movement. **(B)** the total path length moved by the mice. **(C)** resting time. **(D)** the number of passing boxes. **(E)** grip strength. *N* = 8 for in each group. ^ns^P>0.05, ***p < 0.001 and ****p < 0.0001 vs. the PG group.

Compared with the CG group, the trajectories of the open field test showed the trajectories were simple, the activity in the middle area was reduced, the total moved path length was significantly reduced (Figures 2A,B, *p* < 0.0001), and the rest time was significantly prolonged (Figure 2C, *p* < 0.0001), the number of crossing boxes was significantly reduced in the PG group (Figure 2D, *p* < 0.0001); Compared with the PG group, the movement trajectory of the ApoAI MP group was more complex (Figure 2A), the activity in the middle area increased, and the motor control ability was improved. The total moved path length was significantly increased (Figures 2A,B, *p* < 0.0001), the resting time was significantly shortened (Figure 2C, *p* < 0.0001), and the number of crossing boxes was significantly increased in the HG group (Figure 2D, *p* < 0.0001), which increased with the increase in the concentration of ApoAI MP. No outliers were found in all the groups and subsequent experiments, which may be caused by the limited number in each group. Compared with the PG group, the grip strength increased in the LG group (*p* = 0.0009), and further increased in the MG and HG groups (Figure 2E, *p* < 0.0001). All these results indicate that ApoAI MP can enhance the autonomous behavioral activity of mice.

PD induction significantly affected motor coordination (7 mice (87.5%) fell from the rotating Rota-rod within 2 min in the PG group). ApoAI MP interment improved the motor coordination (6 mice (75.0%) fell from the rotating Rota-rod within 2 min in the LG group, 4 mice (50.0%) fell from the rotating Rota-rot within 2 min in the MG group, and 4 mice (50.0%) fell from the rotating Rota-rod within 2 min in the HG group). In contrast, no mouse fell down from the rotating Rota-rod within 2 min. The functional role of ApoAI MP mediating the motor coordination has been suggested. Further work was needed to identify the effect.

### Apolipoprotein A-I mimetic peptides improved circulating lipid profile in Parkinson’s disease mice

**FIGURE 3:**
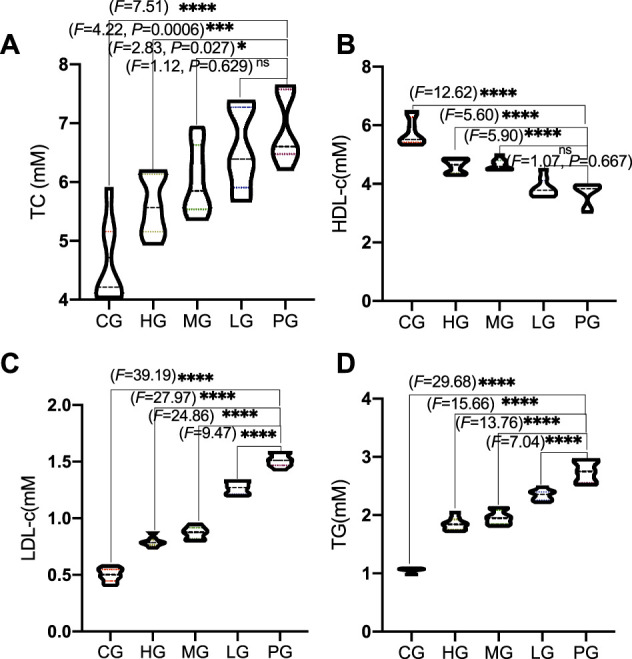
The effects of ApoAI MP on circulating lipid profile. **(A)**, TC. **(B)**, LDL-c. **(C)**, HDL-c. **(D)**, TG. *n* = 8 for in each group_._^ns^ *P*>0.05, *p < 0.05, ***p < 0.001 and ****p < 0.0001 vs. the PG group.

Compared with the CG group, circulating profile of TC (Figure 3A) and LDL-c (Figure 3B) were significantly increased in the mice from the PG group (*p* < 0.0001). HDL-c levels were increased in the mice from the HG group and TG levels were reduced in the mice from HG group when compared with the PG group (Figures 3C,D, *p* < 0.0001). Circulating profile of TC (Figure 3A) and LDL-c (Figure 3B) were significantly reduced in the mice from the ApoAI MP groups and affected in a dose-dependent way. HDL-c levels were increased in the mice from the ApoAI MP groups and affected in a dose-dependent way (Figure 3C) (*p* < 0.05). TG levels were reduced in the mice from the ApoAI MP groups and affected in a dose-dependent way (Figure 3D, *p* < 0.0001). These results suggest that ApoAI MP improved circulating lipid profile in PD mice by increasing HDL-c level and reducing the levels of TC, TG and LDL-c *in vivo*. However, no significant weight loss was found during 2 weeks after MPTP treatment (*p* > 0.05)_._

### Apolipoprotein A-I mimetic peptides enhanced antioxidant properties of Parkinson’s disease mouse model

**FIGURE 4:**
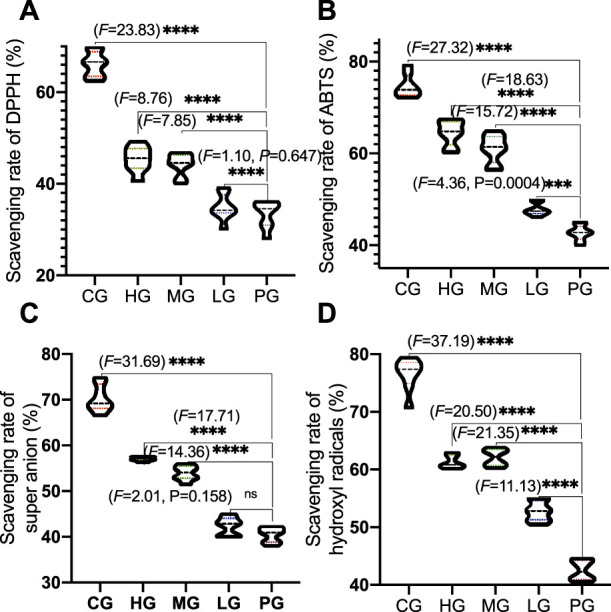
Radical scavenging activities of ApoAI MP among different groups. **(A)**, DHHP. **(B)**, ABTS. **(C)**, superoxide anion. **(D)**, hydroxyl radical. *N* = 8 for in each group. ^ns^P>0.05, ***p < 0.001 and ****p < 0.0001 vs. the PG group.

The antioxidant properties of ApoAI MP include scavenging of ABTS, DPPH, hydroxyl radicals and superoxide anion. Compared with the CG group, the scavenging rates of ABTS (Figure 4A), DPPH (Figure 4B), hydroxyl radicals (Figure 4C) and superoxide anion (Figure 4D) were significantly lower than those in the PG group (*p* < 0.0001), suggesting that AD model establishment increased the oxidative stress by increasing the levels of ABTS, DPPH, hydroxyl radicals and superoxide anion. The scavenging rate of ApoAI MP increased with the increase in concentration. The scavenging rate of the four free radicals in the high concentration ApoAI MP group was significantly higher than that in the LG group (*p* < 0.0001). These results suggest that ApoAI MP reduces the oxidative stress of PD mouse model in a concentration-dependent way, which may be due to the fact that high concentrations of peptides can bind more active sites of free radicals. ApoAI MP reduces the oxidative stress of PD mouse model by scavenging ABTS, DPPH, hydroxyl radical and superoxide anion.

**FIGURE 5:**
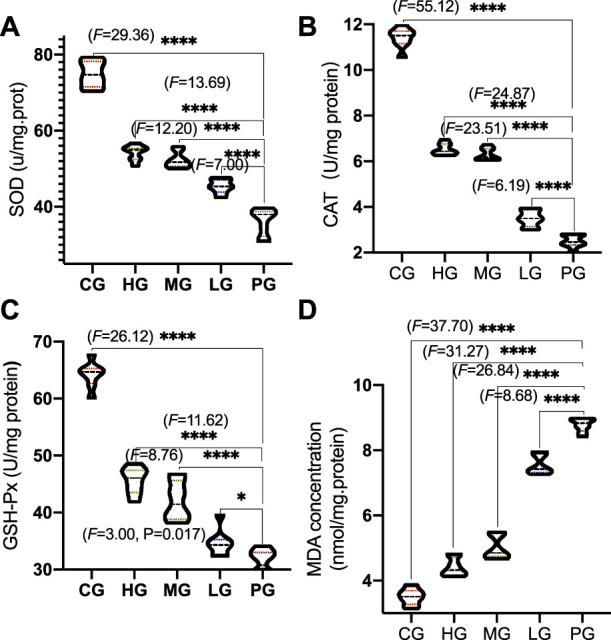
Antioxidant activities of ApoAI MP among different groups. **(A)**, SOD. **(B)**, CAT. **(C)**, GSH-Px. **(D)**, MDA. *n* = 8 for in each group. *p < 0.05, and ****p < 0.0001 vs. the PG group.

Compared with the CG group, the activities of SOD (Figure 5A), CAT (Figure 5B) and GSH-Px (Figure 5C, *p* < 0.0001) was significantly decreased (*p* < 0.01) in the PG group, while the content of MDA was significantly increased (Figure 5D, *p* < 0.0001); Compared with the PG group, ApoAI MP treatment increased the activities of SOD (Figure 5A, *p* < 0.05), CAT (Figure 5B) and GSH-Px (Figure 5C) except of the LG group and reduced MDA concentration (Figure 5D, *p* < 0.0001). It shows that ApoAI MP can increase the level of antioxidant factors and reduce the peroxidation products in a dose-dependent way.

**FIGURE 6:**
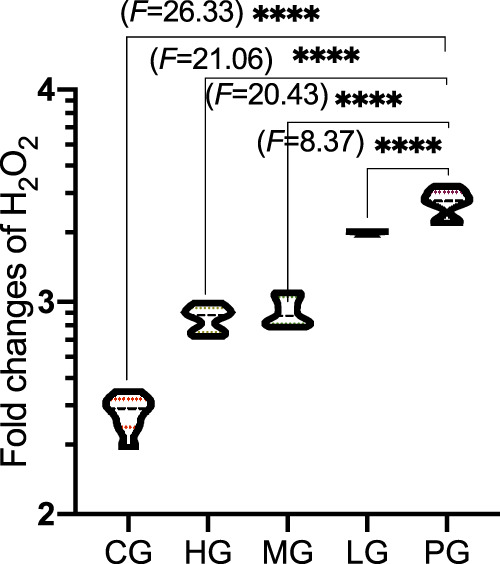
The effects of ApoAI MP on the production of H_2_O_2_ concentration. *N* = 8 for in each group. ****p < 0.0001 vs. the PG group.

Compared with the CG group, the levels of H_2_O_2_ (Figure 6, *p* < 0.0001) was significantly decreased in the PG group. Compared with the PG group, ApoAI MP treatment reduced the levels of H_2_O_2_ (Figure 6, *p* < 0.0001). ApoAI MP reduced the levels of H_2_O_2_ in a dose-dependent way.

### Apolipoprotein A-I mimetic peptides increased anti-inflammatory properties of Parkinson’s disease mouse model

**FIGURE 7:**
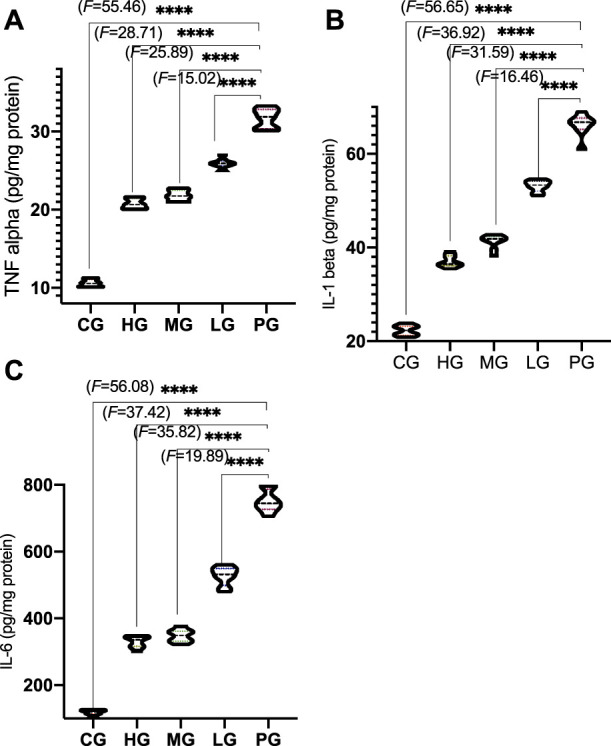
Anti-inflammatory activities of ApoAI MP among different groups_._ **(A)**_,_ TNF-α_._ **(B)**_,_ IL-1β. **(C)**,IL-6_._ *N* = 8 for in each group. ****p < 0.0001 vs. the PG group.

Compared with the CG group, the levels of _TNF-α_ (Figure 7A, *p* < 0.0001), _IL-1β_ (Figure 7B, *p* < 0.0001), and IL-6 (Figure 7C, *p* < 0.0001) was significantly decreased n the PG group. Compared with the PG group, ApoAI MP treatment reduced the levels of _TNF-α_ (Figure 7A, *p* < 0.0001), _IL-1β_ (Figure 7B, *p* < 0.0001), and IL-6 (Figure 7C, *p* < 0.0001). ApoAI MP reduced the levels of _TNF-α_ (Figure 7A, *p* < 0.05), _IL-1β_ (Figure 7B, *p* < 0.0001), and IL-6 (Figure 7C, *p* < 0.0001) in a dose-dependent way. The results suggest that ApoAI MP can reduce the secretion of inflammatory factors.

### Apolipoprotein A-I mimetic peptides rescued striatal dopamine and serotonin levels in the mouse Parkinson’s disease model

**FIGURE 8:**
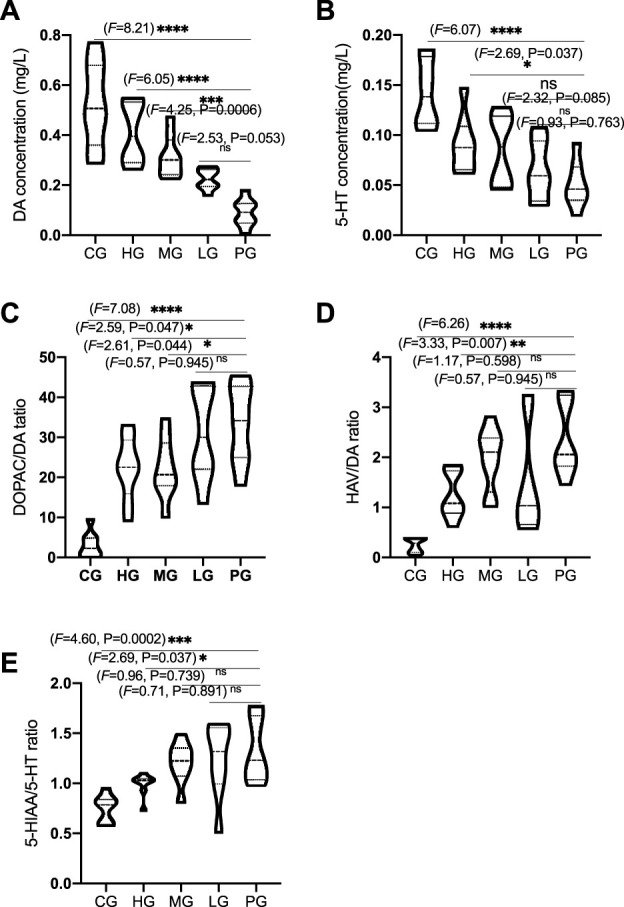
ApoAI MP therapy of PD mice mitigates MPTP-induced reduction of neurotransmitters in the mice striatum. **(A)**, DA concentration. **(B)**, 5-HT concentration. **(C)** and **(D)**, the ratios of DOPAC/DA and HVA/DA represent the levels of DA turnover. **(E)**, 5-HT turnover (the ratio of 5-HIAA/5-HT). *N* = 8 for in each group. ^ns^P>0.05, *p < 0.05, **p < 0.01, ***p < 0.001 and ****p < 0.0001 vs. the PG group.

To evaluate protective impacts on PD, striatal neurotransmitters, DA and 5-HT, DOPAC, HVA, 5-HIAA, were measured by using HPCL. PD establishment considerably reduced the DA concentration when compared with the CG group (Figure 8A, *p* < 0.0001). ApoAI MP treatment significantly increased DA concentration in the mice from the HG (Figure 8A, *p* < 0.0001) and MG (Figure 8A, *p* < 0.001) groups but no significant changes in the mice from the LG group (Figure 8A, *p* > 0.05). Similarly, PD establishment considerably reduced the 5-HT concentration when compared with the CG group (Figure 8B, *p* < 0.0001). ApoAI MP treatment significantly increased 5-HT concentration in the mice from the HG (Figure 8B, *p* < 0.05) but no significant changes in the mice from the MG and LG groups (Figure 8B, *p* > 0.05). In contrast, PD establishment considerably increased the ratios of DOPAC/DA (Figure 8C, *p* < 0.0001) and HVA/DA (Figure 8D, *p* < 0.0001, represent the levels of DA turnover) and 5-HIAA/5-HT (Figure 8E, *p* < 0.0001, represent the levels of 5-HT turnover) when compared with those in the mice from the CG group. ApoAI MP treatment significantly reduced the ratios of DOPAC/DA (Figure 8C, *p* < 0.05) and HVA/DA (Figure 8D, *p* < 0.001) and 5-HIAA/5-HT (Figure 8E, *p* < 0.05) in the HG group when compared with those in the mice from the PG group but no significant changes in the mice from the LG group (*p* > 0.05). These results suggest that ApoAI MP contribute to the improvement of brain neurotransmitters by affecting DA and 5-HT and their turnover.

## Discussion

ApoAI MP can enhance the autonomous behavioral activity of mice by increasing the total moved path length and number of crossing boxes, and reducing the resting time (Figure 2), suggesting that ApoAI MP is a potential drug in improving symptoms of PD models. Oral administration of ApoAI MP also shows protective functions against cognitive deficits and memory decline in Alzheimer’s disease (AD) (Handattu et al., 2009).

**FIGURE 9:**
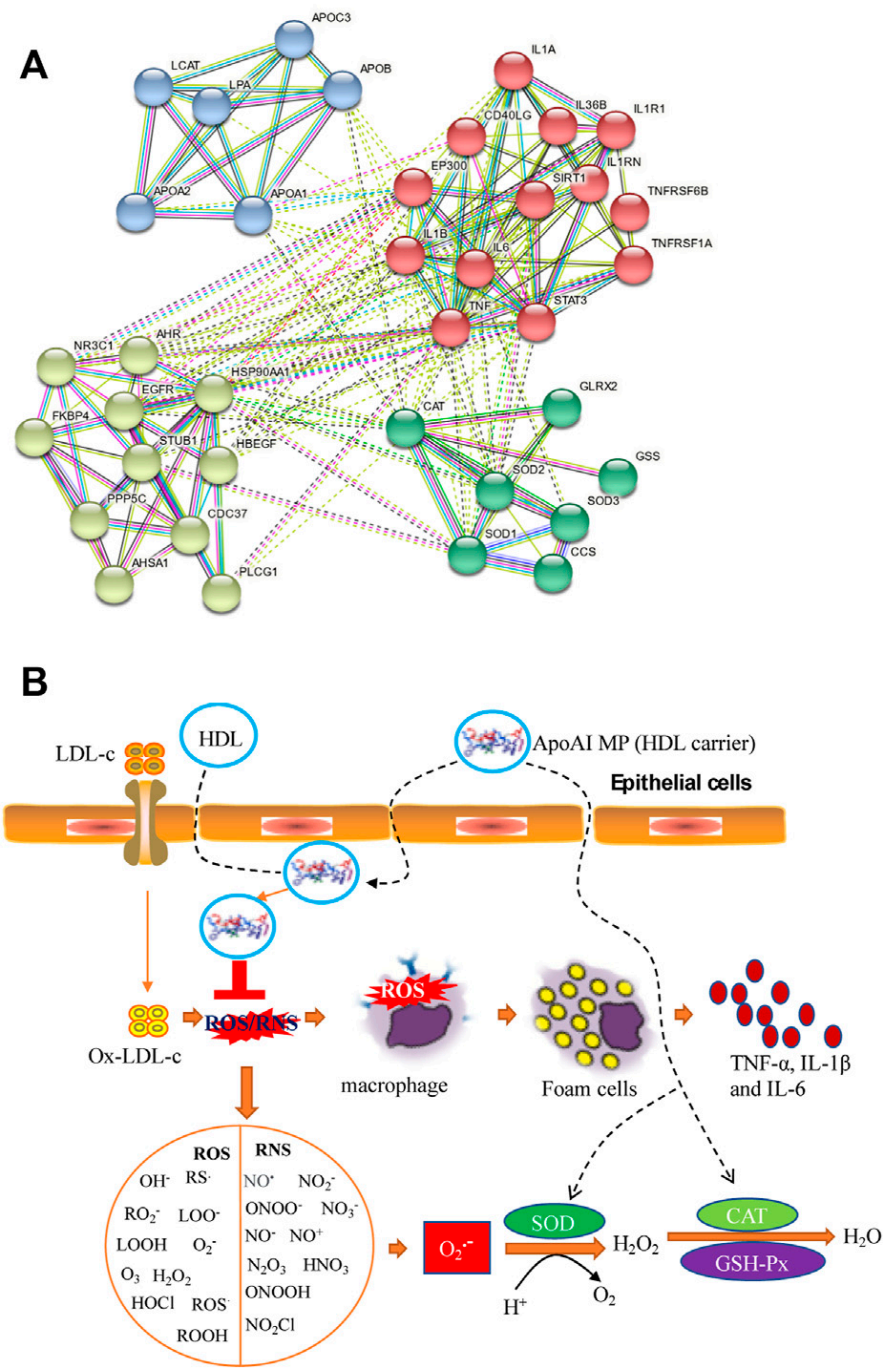
The possible mechanism for the functional role of ApoAI MP in the prevention of PD progression. **(A)**, the interactions among ApoAI, inflammatory cytokines and antioxidants. **(B)**, the possible pathway for the functional role of ApoAI MP. ROS and RNS produced by lipid peroxidation and inflammatory situation. ROS are grouped as oxygen-free radicals and RNS are grouped as nitrogen-free radicals, including hydroxyl radical (·OH), superoxide (O_2_·−), peroxyl radicals (RO_2_·), sulfonyl radicals (ROS·), thiyl radicals (RS·), single oxygen (O_2_•), hydrogen peroxide (H_2_O_2_), hydrogen peroxide and hydroperoxides (ROOH), ozone (O_3_), hypochloride (HOCl), nitric oxide (NO·), peroxynitrite (ONOO^−^), peroxynitrous acid (ONOOH), nitronium (NO_2_^+^), dinitrogen dioxide (N_2_O_2_), and related nitrogen oxides (N_2_O_3_ etc.).

The present findings indicate that ApoAI MP treatment can improve circulating lipid profile in the PD mice model by increasing HDL-c level and reducing the levels of TC, TG and LDL-c (Figure 3). Mendelian randomization analysis shows the inverse association of TC, LDL-C, and TG with PD risk (Fang et al., 2019). Other work suggests that HDL-C levels are lower in the PD model compared with corresponding controls (Choe et al., 2021). ApoAI MP may show neuroprotective effects on PD risk by improving lipid profiles. String protein interaction analysis shows that ApoAI MP may control oxidative stress by activating CAT activity, which is involved with the activation of SOD1, SOD2 and SOD3 (Figure 9A). ApoAI MP intervention control inflammatory responses by inactivating IL-1β, IL-6 and TNF-α responses (Figure 9A). HDL as ApoAI MP carrier counterbalance the inflammatory responses caused by oxidized LDL-c via inhibiting intracellular ROS rise (Figure 9B). SOD, CAT and GSPx, have been recognized as important antioxidants against ROS where SOD scavenges O2—while CAT/GSPx scavenge H_2_O_2_. ApoAI MP reduces oxidative stress by increasing the levels of these antioxidants (Figure 9B).

ApoAI MP reduces the oxidative stress of PD mouse model by scavenging ABTS, DPPH, hydroxyl radical and superoxide anion (Figure 4). ABTS and DPPH are main components of free radicals and can cause damage in PD patients and the antiradical activity against such free radicals will be an efficient protective way for PD patients (Anastassova et al., 2019). The partial reduction of oxygen (superoxide anion, hydroxyl radical) can cause neural toxicity for PD patients and these free radicals clearance is a new therapeutic strategy in PD36.

Although the etiology of PD is not fully understood, oxidative stress (Trist et al., 2019) and inflammatory responses (Gao et al., 2022) are important risks links leading to PD disease. The present findings demonstrated that ApoAI MP increased antioxidant properties of PD mouse model by increasing the activities of SOD, CAT and GSH-Px and reducing the content of MDA (Figure 5). During PD, the activities of SOD and the CAT and GSH-Px of the antioxidant defense system are relatively weak, and the improvement of their activities is an important approach in the prevent of PD progression and development (Wang et al., 2018; Chen et al., 2021). Monoamine oxidase promotes the generation of H_2_O_2_, which increases high levels of oxidative stress state and cause injury in PD patients (Youdim, 2018). GSH-Px can scavenge H_2_O_2_ and catalyze H_2_O_2_ with GSH) to produce H_2_O and GSSG under physiological conditions to avoid the toxic effect of H_2_O_2_ (Song et al., 2021). MDA is one of the final products of polyunsaturated fatty acid peroxidation and a well-established marker of oxidative stress, which is associated with PD injury. The control of MDA is a potential way in the clinical treatment of PD (Tamtaji et al., 2019). On the other hand, excessive ROS and H_2_O_2_ are the key mediator of PD pathogenesis (Weng et al., 2018). ApoAI MP will also show beneficial effects to control PD development by reducing the damage caused by ROS and H_2_O_2_.

The secretion of proinflammatory cytokines such as IL-1β, IL-6 and TNF-α in the brain of PD patients is closely related to the destruction of neurons (Chen et al., 2018). In a model of PD induced by bacterial endotoxin lipopolysaccharide, DA neurons exhibited progressive loss, also suggesting an important role of inflammation in the degeneration of the nigrostriatal pathway (Milde et al., 2021). The present result is consistent with the previous report that ApoAI MP decrease the levels of inflammatory cytokines IL-1β and TNFα in the C57BL/6 mice (Figure 7) (De Lazzari et al., 2018). Therefore, it is critical to control three proinflammatory cytokines (IL-1β, IL-6 and TNF-α) to prevent PD progression (Cheng et al., 2021).

MPTP is often used to induce mouse model of PD by reducing the levels of neurotransmitters such as striatal DA and 5-HT (Luchtman et al., 2009). ApoAI MP improve PD by preventing the decrease of striatal DA and 5-HT levels, and their turnover caused by MPTP. The serotonergic system plays an important role in is cognitive behavior. Striatal DA Dopamine depletion is closely associated with cognitive deficits in early-stage PD (Chung et al., 2018). The reduction in the striatal 5-HT levels will cause mood disturbances and cognitive impairments of PD (Miguelez et al., 2014). The present findings powerfully support that ApoAI MP may be potential natural biomedicine for preventing PD development via improvement of brain inflammation and neurotransmitters.

There were some limitations in the present work. Oral administration of ApoAI MP improves cognitive function and reduces amyloid burden in a mouse model with Alzheimer’s disease and shows protective functions against cognitive deficits and memory decline in neurodegenerative diseases (Handattu et al., 2009). Amyloid burden is also associated with the PD development and was not explored in the present study (Lim et al., 2019). PD is the very common neurodegenerative diseases characterized by aggressive loss of dopaminergic neurons in the substantia nigra and accumulation of oxidative stress (Krashia et al., 2019). Such linkage was not measured either. It is important to consider the effect of behavior alone, and also perhaps correlations could be conducted between behavioral and molecular endpoints. Unfortunately, the number in each group (*n* = 8) was very limited and the effects of behavioral test on molecular endpoints were not explored. Just for the case, the adjustment based on the effects of behavioral test was not considered. There are very important differences in lipid metabolism between mice and humans. The effects of ApoAI MP on lipid metabolism of human beings remain unclear. Therefore, more studies are needed to address these important issues in the future.

## Conclusion

In this study, ApoAI MP was used to explore its functional role in the PD model, showing that ApoAI MP can enhance the autonomous activity ability and improve the movement disorder, and motor coordination of PD mice. The test results of oxidative stress indicators showed that ApoAI MP at 1,000 μg/ml had a significant effect on ABTS, DPPH, hydroxyl radical and superoxide anion scavenging rates, indicating that ApoAI MP is a strong antioxidant peptide. ApoAI MP can increase SOD, CAT, GSH-Px activity, up-regulate GSH content, and reduce the level of malondialdehyde, a marker of lipid peroxidation; and it was suggested that the therapeutic mechanism of ApoAI MP on PD may be related to alleviating oxidative stress and reducing the damage of ROS and H_2_O_2_. Finally, ApoAI MP may improve the symptoms of PD model by improving neurotransmitters. This study provides a new strategy and idea for the clinical treatment of PD, but it still needs to be further studied in order to provide more scientific basis.

## Data Availability

The original contributions presented in the study are included in the article/supplementary material, further inquiries can be directed to the corresponding author.
